# Resveratrol improves motor function in patients with muscular dystrophies: an open-label, single-arm, phase IIa study

**DOI:** 10.1038/s41598-020-77197-6

**Published:** 2020-11-25

**Authors:** Kentaro Kawamura, Shinobu Fukumura, Koki Nikaido, Nobutada Tachi, Naoki Kozuka, Tsugumi Seino, Kingya Hatakeyama, Mitsuru Mori, Yoichi M. Ito, Akiyoshi Takami, Shiro Hinotsu, Atsushi Kuno, Yukihiko Kawasaki, Yoshiyuki Horio, Hiroyuki Tsutsumi

**Affiliations:** 1 Department of Pediatrics, Sapporo Medical University School of Medicine, Sapporo, 060-8543 Japan; 2 Faculty of Health Science, Hokkaido Chitose College of Rehabilitation, Chitose, 066-0055 Japan; 3 Department of Physical Therapy, Sapporo Medical University School of Health Sciences, Sapporo, 060-8556 Japan; 4 Biostatistics Division, Clinical Research and Medical Innovation Center, Hokkaido University Hospital, Sapporo, 060-8648 Japan; 5 Division of Hematology, Department of Internal Medicine, Aichi Medical University School of Medicine, Nagakute, 480-1195 Japan; 6 Department of Biostatistics, Sapporo Medical University School of Medicine, Sapporo, 060-8556 Japan; 7 Department of Pharmacology, Sapporo Medical University School of Medicine, Sapporo, 060-8556 Japan

**Keywords:** Drug discovery, Diseases, Medical research, Neurology

## Abstract

Muscular dystrophies (MDs) are inherited disorders characterized by progressive muscle weakness. Previously, we have shown that resveratrol (3,5,4′-trihydroxy-trans-stilbene), an antioxidant and an activator of the protein deacetylase SIRT1, decreases muscular and cardiac oxidative damage and improves pathophysiological conditions in animal MD models. To determine whether resveratrol provides therapeutic benefits to patients with MDs, an open-label, single-arm, phase IIa trial of resveratrol was conducted in 11 patients with Duchenne, Becker or Fukuyama MD. The daily dose of resveratrol was 500 mg/day, which was increased every 8 weeks to 1000 and then 1500 mg/day. Primary outcomes were motor function, evaluated by a motor function measure (MFM) scale, muscular strength, monitored with quantitative muscle testing (QMT), and serum creatine kinase (CK) levels. Adverse effects and tolerability were evaluated as secondary outcomes. Despite the advanced medical conditions of the patients, the mean MFM scores increased significantly from 34.6 to 38.4 after 24 weeks of medication. A twofold increase was found in the mean QMT scores of scapula elevation and shoulder abduction. Mean CK levels decreased considerably by 34%. Diarrhoea and abdominal pain was noted in six and three patients, respectively. Resveratrol may provide some benefit to MD patients.

## Introduction

Muscular dystrophies (MDs) are inherited myopathic disorders characterized by progressive muscle weakness and disability^1^. Mutations in a wide range of proteins, including the dystrophin-associated glycoprotein complex, which connects the myofibre cytoskeleton to the extracellular matrix, have been implicated in MDs^1^. Complete loss of the function of dystrophin causes Duchenne muscular dystrophy (DMD), the most common and lethal X-linked myopathy, whereas a partial loss of the function results in Becker muscular dystrophy (BMD), which has a milder phenotype^1^. Mutations in the *FKTN* gene cause Fukuyama congenital muscular dystrophy (FCMD), a fatal and the second most prevalent form in Japan^2^. Although novel therapeutic strategies based on stop codon readthrough or exon skipping have been developed^3,4^, the conventional and reliable medicine for DMD is glucocorticoids, which improve motor function and the quality of life. Glucocorticoids delay the median age of loss of mobility in DMD patients by 2.1–4.4 years^5^, but the medical condition of patients steadily deteriorates under medication. In addition, glucocorticoids have other adverse effects such as cushingoid features, weight gain, behavioural changes and growth delay^5^.

SIRT1, an NAD^+^-dependent protein deacetylase, is a mammalian homologue of Sir2 that prolongs lifespan when overexpressed in yeast, *Caenorhabditis elegans*, and *Drosophila melanogaster*^6^. SIRT1 deacetylates histones, various transcription factors and cytoplasmic proteins and plays critical roles in cell survival by reducing reactive oxygen species (ROS), inhibiting inflammation, and promoting mitochondrial function and autophagy^6,7^. SIRT1 overexpression in the skeletal muscles of *mdx* mice, an animal model of DMD, decreases serum creatine kinase (CK) levels, tissue fibrosis, and myofibril damage and increases their ability to run long distances compared with control *mdx* mice^8^.

Resveratrol (3,5,4′-trihydroxy-trans-stilbene), a natural polyphenol found in grapes and red wine, has been shown to allosterically activate SIRT1^6,7^. δ-Sarcoglycan is a component of the dystrophin glycoprotein complex, the defect of which leads to severe cardiomyopathy in hamsters. We found that SIRT1 induces antioxidative superoxide dismutase 2 (SOD2), and oral administration of resveratrol to δ-sarcoglycan-deficient TO-2 hamsters increases cardiac SOD2 levels, attenuates the deterioration of cardiac function and hypertrophy, and significantly extends the lifespan^9^. Resveratrol administration also improves the pathophysiological manifestations of dystrophin-deficient *mdx* mice. Resveratrol decreases oxidative stress levels in muscles, reduces cardiac and muscular fibrosis, increases the expression levels of myosin heavy chains and troponins, and inhibits cardiac hypertrophy^10–12^. Moreover, long-term administration of resveratrol improves cardiac and muscular function with a decrease in serum CK levels to one-third of those in untreated *mdx* mice^11,12^. Thus, resveratrol could be a useful medicine for treating MD patients. However, to date, there have been no clinical studies investigating this possibility. Because clinical studies show the relatively low toxicity of resveratrol^13^, we planned a pilot phase IIa study in MD patients to investigate its biological activity.

In the present study, we administered resveratrol to patients with DMD, BMD, or FCMD for 24 weeks. Since the diseases had progressed considerably in these patients, motor function, muscular strength, and CK levels were evaluated as primary outcomes. Adverse effects and tolerability were also evaluated as secondary outcomes.

## Results

### Patients and protocol

**Figure 1:**
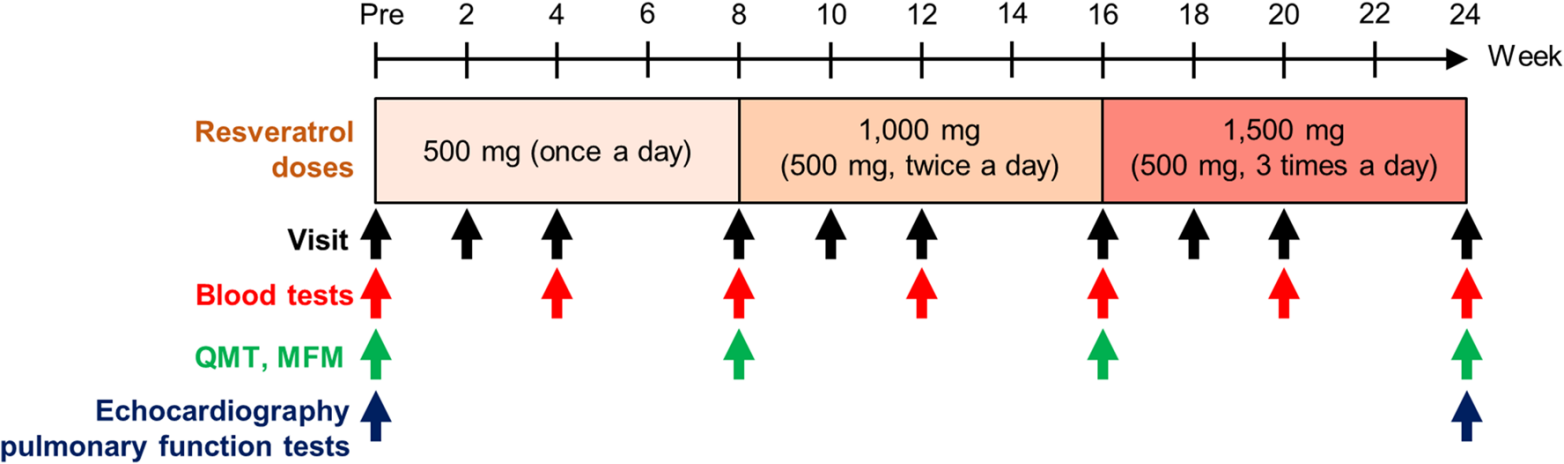
Protocol for resveratrol administration. Daily doses of resveratrol are shown, and visits along with blood and physical examinations are indicated by arrows.

The mean ages of eleven DMD, BMD, and FCMD patients were 21.0 ± 9.7 years (range, 12–39 years), 33.5 ± 8.3 years (range, 23–46 years), and 14.5 ± 2.5 years (range, 12–17 years), respectively, and the mean age of all participants was 24.4 ± 11.1 years (range, 12–46 years) (Table 1). One FCMD patient was female, and the remaining ten participants were male. One DMD patient and three patients with BMD were ambulatory. The other seven patients were unable to walk and needed a wheelchair; therefore, they were assigned a grade of 9 on the Vignos scale^14^. Resveratrol was orally administered daily to all participants at an initial dose of 500 mg/day for 8 weeks (Fig. 1). The dose was then increased every 8 weeks to 1000 and finally to 1500 mg/day, similar to dosages of resveratrol administered to healthy subjects and patients with cancer or metabolic diseases^13^. All patients were assessed for motor function, muscle strength, and blood examinations. Mean scores of muscle function measure (MFM), quantitative muscle testing (QMT), and serum CK levels of participants before resveratrol medication are shown in Table 2.

**Table 1:** Participants characteristics.

	Type	Age, years	Sex	Weight (kg)	Genetic testing	Other diagnostic basis	Vignos scale	Brooke scale
1	Duchenne	12	Male	35.9	Deletion of exon 18		Grade 4	Grade 2
2		14	Male	27.5	Deletion of exons 44–47	Family history	Grade 9	Grade 4
3		17	Male	45.8	Deletion of exon 11		Grade 9	Grade 6
4		23	Male	47.7	Not available	Biopsy	Grade 9	Grade 6
5		39	Male	49.8	Not available	Family history	Grade 9	Grade 6
6	Becker	23	Male	64.0	Deletion of exons 45–48		Grade 2	Grade 1
7		31	Male	71.0	Deletion of exon 3		Grade 9	Grade 6
8		34	Male	64.5	No copy number variation	Biopsy	Grade 3	Grade 1
9		46	Male	64.0	Deletion of exons 45–48	Biopsy	Grade 6	Grade 2
10	Fukuyama	12	Female	26.9	Heterozygous for the founder haplotype	Brain MRI	Grade 9	Grade 6
11		17	Male	22.6	Homozygous for the founder haplotype	Biopsy	Grade 9	Grade 6

**Table 2:** Mean MFM scale and QMT scores of participants before resveratrol administration.

	Type of muscular dystrophy									Total (N = 11)		
	Duchenne (N = 5)			Becker (N = 4)			Fukuyama (N = 2)					
	Number (%)	Range	Mean (SD)	Number (%)	Range	Mean (SD)	Number (%)	Range	Mean (SD)	Number (%)	Range	Mean (SD)
MFM scale	5 (100)	2–63	22.2 (21.5)	4 (100)	38–85	64.5 (16.9)	2 (100)	2–10	6	11 (100)	2–85	34.6 (29.3)
QMT pinch (N)	5 (100)	0.5–2.4	11.1 (0.7)	4 (100)	0.3–4.9	3.8 (2.1)	2 (100)	0.1–0.5	0.3	11 (100)	0.1–4.9	1.9 (2.0)
QMT grip (kgf)	3 (60)	0.8–4.8	2.3 (1.8)	4 (100)	1.3–25.5	12.7 (9.5)	0 (0)	–	–	7 (64)	0.8–25.5	8.3 (8.9)
QMT scapula elevation (kgf)	4 (80)	0.9–8.6	3.3 (3.1)	4 (100)	5.3–6.8	6.0 (0.6)	2 (100)	1.4–1.4	1.4	10 (91)	0.9–8.6	4.0 (2.7)
QMT shoulder abducttion (kgf)	1 (20)	–	3.0 (–)	4 (100)	3.5–6.2	4.7 (1.0)	0 (0)	–	–	5 (45)	3.0–6.2	4.4 (1.1)
QMT elbow flexion (kgf)	1 (20)	–	1.6 (–)	4 (100)	0.6–6.2	5.1 (3.0)	1 (50)	–	1.1	6 (55)	0.6–6.2	3.9 (3.1)
QMT elbow extension (kgf)	1 (20)	–	2.8 (–)	4 (100)	3.1–9.2	5.7 (2.5)	0 (0)	–	–	5 (45)	2.8–9.2	5.1 (2.5)
QMT hip flexion (kgf)	1 (20)	–	5.2 (–)	4 (100)	4.3–10.1	7.7 (2.2)	0 (0)	–	–	5 (45)	4.3–10.1	7.2 (2.2)
QMT knee extension (kgf)	2 (40)	2.0–2.5	5.2 (–)	4 (100)	1.3–11.4	6.0 (3.9)	0 (0)	–	–	6 (55)	1.3–11.4	4.8 (3.6)
QMT ankle dorsiflexion (kgf)	1 (20)	–	5.2 (–)	4 (100)	3.2–7.9	5.4 (2.2)	1 (50)	–	1.4	6 (55)	1.4–7.9	4.4 (2.4)
Creatine kinase (unit/l)	5 (100)	200–9518	2522 (3518)	4 (100)	431–2807	1601 (1020)	2 (100)	840–1981	1411	11 (100)	200–9518	1985 (2512)

### Motor function

**Figure 2:**
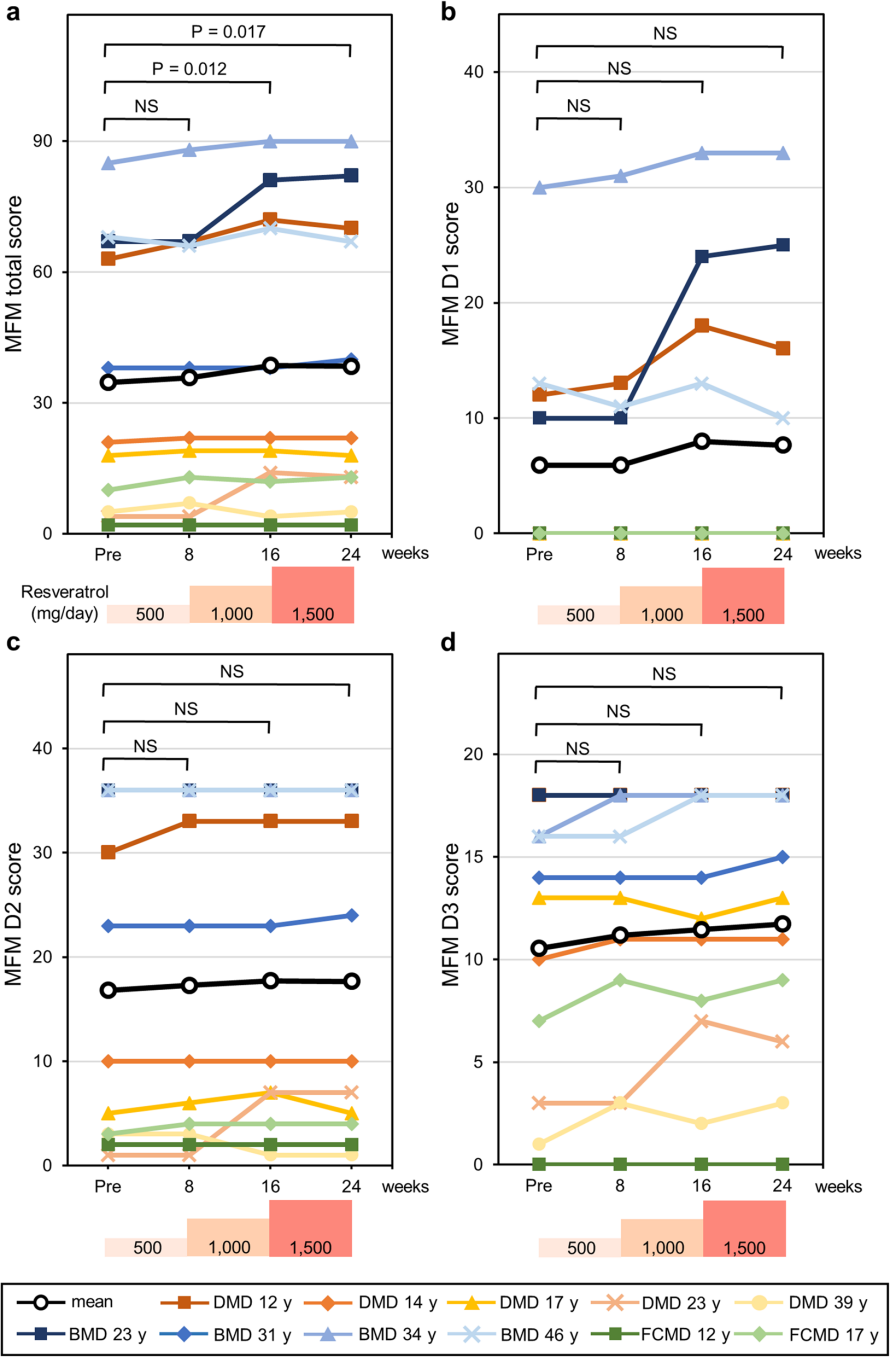
Motor function scores following resveratrol administration. (**a**) Total score, (**b**) D1 sub-score in standing position and transfers, (**c**) D2 sub-score in axial and proximal motor function, and (**d**) D3 sub-score in distal motor function of each patient are measured with the MFM scale before (Pre) and at 8, 16, and 24 weeks. Black lines show mean values. Average values at each visit were compared with those before medication and were analysed using ANOVA. Post hoc analysis was performed using Dunnett’s test. P values < 0.05 are indicated. NS: not significant.

The MFM scale is a sensitive and reliable method for evaluating motor function in neuromuscular diseases, including muscular dystrophy^15^. Patients’ motor functions were evaluated by the MFM scale scores on 32 items in three dimensions. The mean MFM total score of all participants before resveratrol administration was 34.6 ± 29.3 (range, 2–85). MFM scores after 16 and 24 weeks of medication were 38.5 ± 31.7 (range, 2–90) (p = 0.012) and 38.4 ± 31.3 (range, 2–90) (p = 0.017), respectively (Fig. 2a). Thus, long-term administration of resveratrol significantly increased MFM scores by 10%. Sub-score analyses of D1, D2, and D3 showed increasing tendencies, but these did not meet the threshold of statistical significance (Fig. 2b–d), suggesting that resveratrol does not improve muscle function specifically but instead affects the whole body. The Brooke and Vignos scale grades did not change during this study.

### Muscle strength

**Figure 3:**
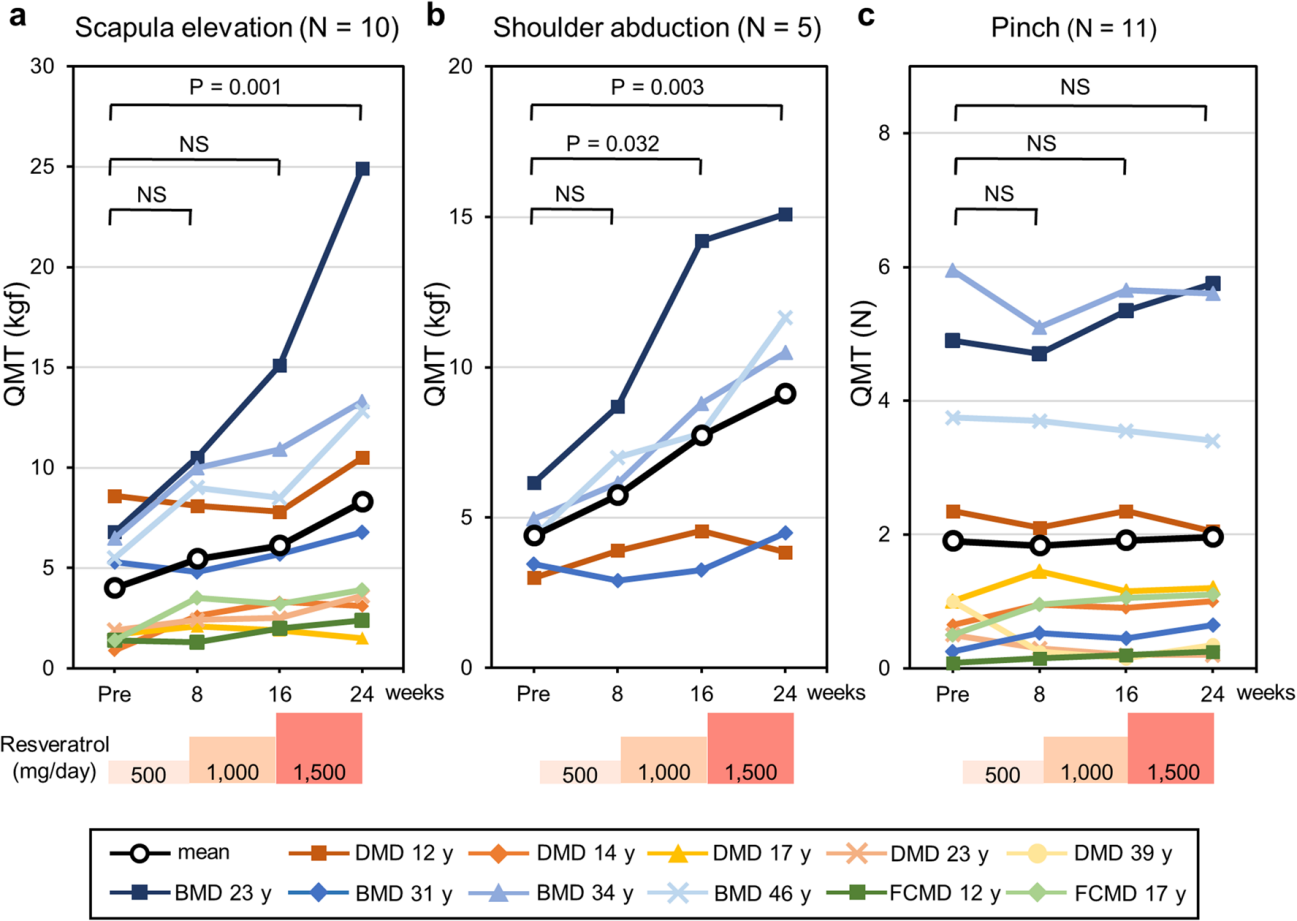
Muscle strength following resveratrol administration. (**a**–**c**) Time course of muscle strength for (**a**) scapula elevation (N = 10), (**b**) shoulder abduction (N = 5), and (**c**) pinch (N = 11) measured by quantitative muscle testing (QMT) using a hand-held dynamometer and pinch strength metre before (Pre) and 8, 16, and 24 weeks after resveratrol administration. The daily dose of each patient is also shown, except that one patient with FCMD (FCMD 12 y) received 1000 instead of 1500 mg/day. Black lines show mean values. Average values at each visit were compared with those before medication and were analysed using ANOVA. Post hoc analysis was performed using Dunnett’s test. P values < 0.05 are indicated. NS: not significant.

To determine whether muscle strength was affected by resveratrol, we evaluated the muscle strength of each patient. Strength was monitored with quantitative muscle testing (QMT), which is a reliable method for evaluating the muscle strength of ambulatory and non-ambulatory MD patients^16–18^. Nine different QMT scores were measured, but in some patients, certain items could not be scored due to their severe muscle weakness or contracture. For example, a 39-year-old patient with DMD could be evaluated only for pinch strength. The monitored activities and number of patients assessed were as follows: scapula elevation, 10; shoulder abduction, 5; pinch, 11; elbow extension, 5; elbow flexion, 6; hip flexion, 5; knee extension, 6; ankle dorsiflexion, 6; and grip, 7 (Table 2, Fig. 3, Supplementary Fig. S1). Scapula elevation was measured in ten participants, including three non-ambulatory patients. The administration of resveratrol for 24 weeks increased the mean QMT score of scapula elevation from 4.0 ± 2.7 kg-force (kgf) (range, 0.9–8.6 kgf) to 8.3 ± 6.9 kgf (range, 1.5–24.9 kgf) (p = 0.001) (Fig. 3a), while the mean shoulder abduction strength measured in one DMD patient and four patients with BMD increased from 4.4 ± 1.1 kgf (range, 3.0–6.2 kgf) to 9.1 ± 4.3 kgf (range, 3.9–15.1 kgf) (p = 0.003) (Fig. 3b). QMT scores of elbow extension, elbow flexion, hip flexion, knee extension, and ankle dorsiflexion in some patients were markedly improved by resveratrol, but the differences were not significant (Supplementary Fig. S1a–e). Resveratrol had no effect on pinch strength between the thumb and forefinger (Fig. 3c) or on grip strength (Supplementary Fig. S1f).

### Serum CK levels

**Figure 4:**
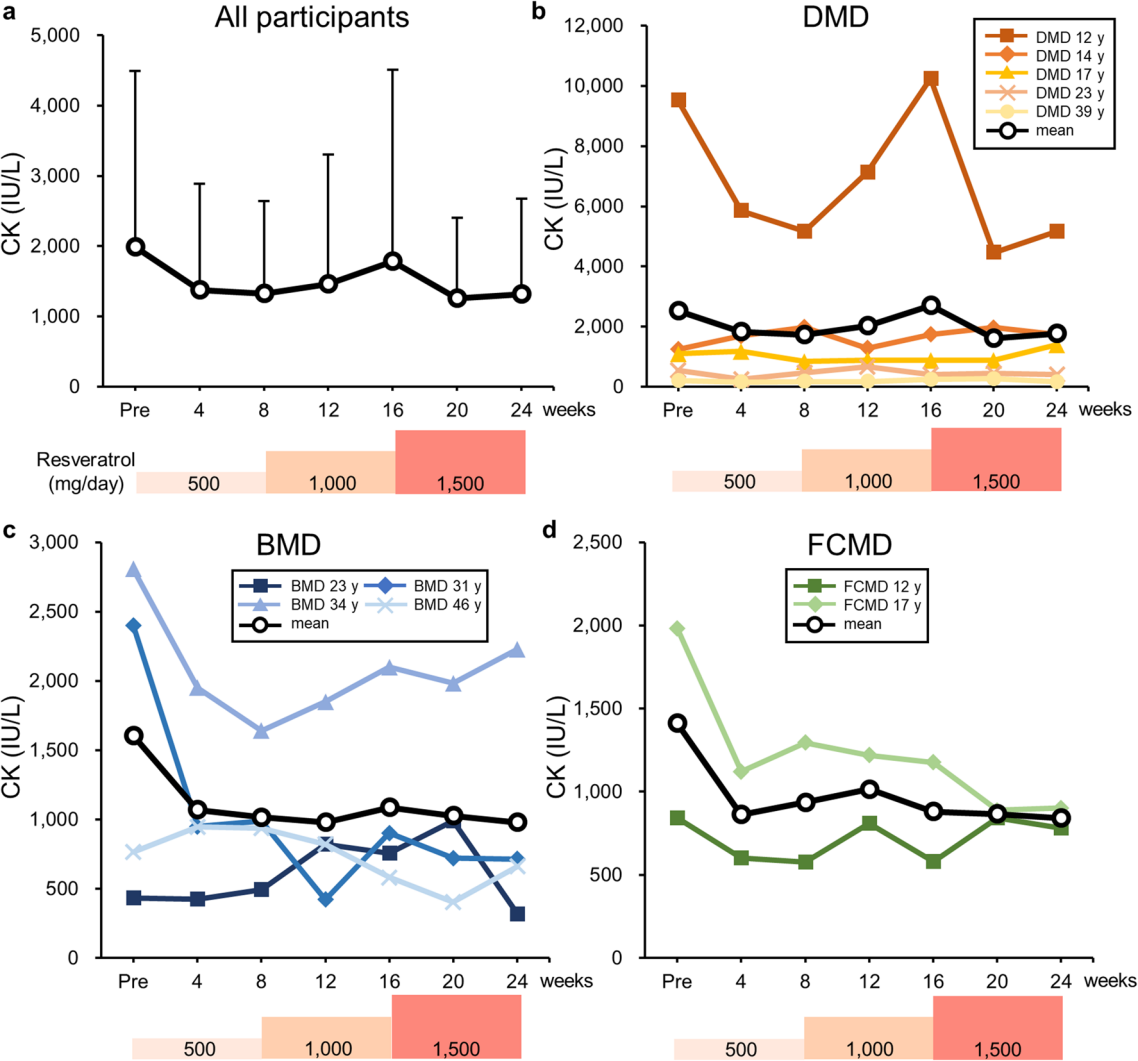
Serum creatine kinase (CK) levels following resveratrol administration. (**a**) Mean serum CK levels of all participants with standard deviation. (**b–d**) Change in CK activity in each patient with (**b**) Duchenne, (**c**) Becker, or (**d**) Fukuyama congenital muscular dystrophy. Black lines show the mean CK levels.

Serum CK levels in patients were examined once every 4 weeks (Fig. 4a–d). The mean CK levels were 1985 ± 2511 IU/L (range, 200–9518 IU/L) before resveratrol administration and 1315 ± 1357 IU/L (range, 166–5167 IU/L) after 24 weeks of medication. Thus, a 34% decrease in mean CK levels was achieved by resveratrol, but the difference was not statistically significant (p = 0.148) (Fig. 4a).

### Serum resveratrol concentrations

**Figure 5:**
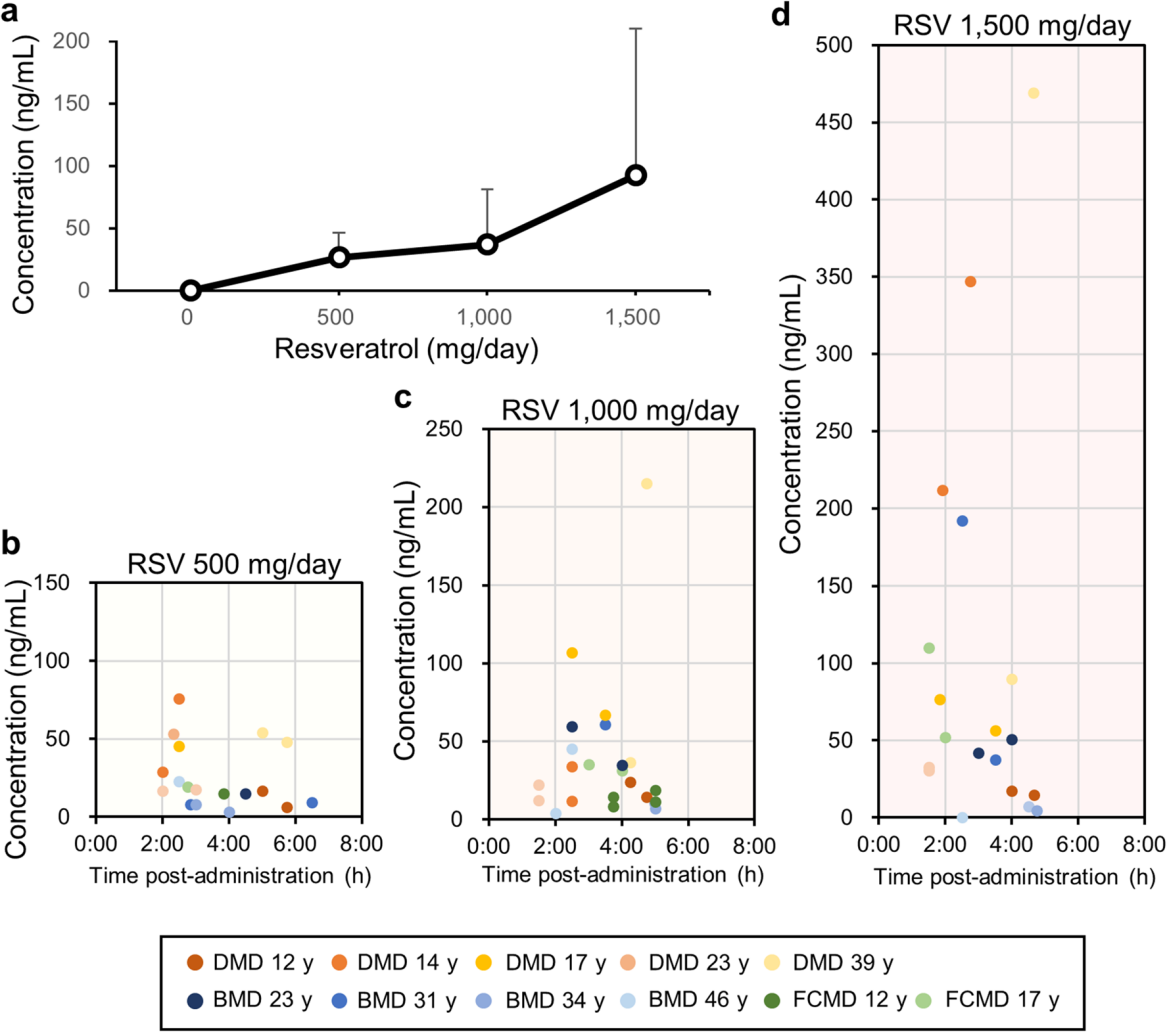
Serum resveratrol concentration at doses of 500, 1000, and 1500 mg/day. (**a**) Mean serum resveratrol levels of all participants with standard deviation (N = 11). (**b**–**d**) Serum resveratrol level and time from resveratrol ingestion to blood drawing in each patient are shown by a dot. RSV: resveratrol.

Serum resveratrol concentrations in all patients were examined every 4 weeks. Levels of serum resveratrol increased in a dose-dependent manner, and the mean resveratrol levels at doses of 500, 1000, and 1500 mg/day were 26.4 ± 20.0 ng/mL (range, 2.6–75.9 ng/mL), 36.9 ± 44.7 ng/mL (range, < 1.0–215.0 ng/mL), and 92.2 ± 117.7 ng/mL (range, < 1.0–469.0 ng/mL), respectively (Fig. 5a). Serum resveratrol concentration and the time interval for blood draw after ingestion in each patient are shown in Fig. 5b–d.

### Adverse events

Grade 1 diarrhoea was noted in four patients (Table 3). Grade 2 diarrhoea was found in two patients administered 1500 mg/day resveratrol. These two patients needed to reduce the dosage from 1500 to 1000 mg/day. One patient resumed a dose of 1500 mg/day after several days, whereas the other patient (FCMD 12 y) was maintained at the dose of 1000 mg/day. Grade 1 abdominal pain was observed in three patients. Two cases of upper respiratory tract infection and one case of pneumonia were observed during the study. Among them, two patients were temporarily hospitalized but left the hospital soon afterwards. During hospitalization, they continued to take resveratrol on schedule. Transient alopecia, erythema multiforme, and acneiform rash were observed in one patient each. All participants continued to take resveratrol for 24 weeks. Resveratrol did not affect blood markers of liver and renal function (Supplementary Table S1).

**Table 3:** Adverse events following resveratrol administration.

Symptom	Grade 1	Grade 2	Grade 3	Grade 4	Grade 5	Total (%)
Diarrhoea	4	2				6 (54.5)
Abdominal pain	3					3 (27.3)
Upper respiratory infection		1	1			2 (18.2)
Lung infection			1			1 (9.1)
Alopecia	1					1 (9.1)
Erythema multiform	1					1 (9.1)
Rash acneiform	1					1 (9.1)

The numbers of patients are indicated.

## Discussion

Resveratrol has been used in humans, and previous data show that its toxicity is limited^13^. Since resveratrol ameliorated pathology and improved the function of MD animal models, we planned this study as a pilot study.

Resveratrol improved motor function and muscle power in the proximal muscles of participants. However, the distal muscles of patients were unaffected (Fig. 3c, Supplementary Fig. S1f). One patient with BMD (BMD 23 y) experienced relief from muscle pain during resveratrol administration, which may have contributed to the improvement in motor function. The observed increase in patients’ muscle strength following resveratrol treatment was consistent with findings in *mdx* mice^10,12^, in which approximately fivefold increases in slow-fibre-type myosin heavy chain and troponin gene transcripts were detected in the biceps femoris muscles^10^. Resveratrol binds the N-terminal region of the SIRT1 protein, resulting in the allosteric activation of SIRT1 activity^7^. SIRT1 deacetylates and activates peroxisome proliferator-activated receptor-γ coactivator 1α (PGC-1α), which switches muscle types from fast to slow fibres^6,7,19^. Indeed, transgenic mice overexpressing PGC-1α^19^ or SIRT1^8^ show muscle type conversion from fast- to slow-twitch fibres. PGC-1α transgenic mice also show increased resistance to muscular fatigue^19^. Thus, the beneficial effects of resveratrol on the muscles of MD patients may involve PGC-1α activation by SIRT1. In *mdx* mice, serum CK levels were decreased by the overexpression of SIRT1 in the muscles^8^ and by the long-term administration of resveratrol^12^. Recently, we have shown that skeletal muscle-specific SIRT1-knockout mice have a fragile muscle cell plasma membrane and that SIRT1 is involved in membrane resealing after cell injury^20^. In the present study, mean CK levels were decreased by resveratrol, although the change was non-significant. We noted that patients with low serum CK levels, i.e., less than 2000 IU/L, showed little response to resveratrol treatment, which could be caused by the lack of sufficient muscle mass. Resveratrol administration at early disease stages may afford great benefits to MD patients.

Oxidative stress plays a key role in the development of MD pathology^21^. Resveratrol decreases ROS levels by inducing the expression levels of the antioxidant enzyme superoxide dismutase^9^, suppressing superoxide-generating NADPH oxidase^10^, and eliminating damaged mitochondria, a major cellular source of ROS^22^, via mitochondrial autophagy, known as mitophagy^11,12,23,24^. These antioxidative functions of resveratrol are mediated at least in part by the deacetylation and activation of Forkhead box O transcription factors and PGC-1α via SIRT1 activation^6,7^.

Cardiomyopathy and subsequent heart failure is a major cause of death in MD patients. Resveratrol suppresses cardiac hypertrophy and fibrosis in TO-2 hamsters^9^ and *mdx* mice^25^, which may be mediated by the deacetylation and ubiquitin-dependent degradation of transcriptional coactivator p300 via SIRT1 activation^25^. In the present study, we could not detect any improvement in echocardiographic parameters or levels of plasma brain natriuretic peptide (BNP), a marker of cardiomyopathy. This may be because all participants showed normal BNP levels (< 18.4 pg/ml), and only 2 patients had a reduced ejection fraction of less than 50% (Supplementary Table S1). Longer-term administration of resveratrol may be necessary to reveal the cardiac function of resveratrol in MD patients.

Resveratrol is rapidly absorbed and yields peak serum concentration at 1 h post dose^13^. In the present study, the levels of serum resveratrol concentration in MD patients (Fig. 5b–d) were consistent with those in healthy volunteers^13^. Six (54.5%) and three (27.3%) patients had diarrhoea and abdominal pain, respectively (Table 3). These symptoms were transient and tolerable in participants except for the female patient (FCMD 12 y), who suffered from severe diarrhoea at the dose of 1500 mg resveratrol. The weight of the patient was only 26.5 kg, and she could not continue the dose. Similar to our study, Brown et al*.* has reported mild to moderate gastrointestinal symptoms in healthy volunteers who ingested resveratrol at doses of 2500 and 5000 mg/day^26^. Two patients contracted transient grade 3 upper respiratory and lung infections, and one patient suffered from a grade 2 upper respiratory infection. We believe that these events were unrelated to resveratrol since all three patients had repeatedly suffered from respiratory infections before the clinical trial and recovered immediately while taking resveratrol.

We found that administration of resveratrol to *mdx* mice with the highest dose (4 g/kg food) is more effective in increasing myofibre cross-section area than lower doses of 0.4 g and 0.04 g/kg food^12^. However, a moderate dose of resveratrol (0.4 g/kg food) also decreases the number of fibres with central nuclei, the number of fine fibres, and serum creatine kinase levels. Additionally, it significantly improves motor function in *mdx* mice^12^. Resveratrol at 0.4 g/kg food was estimated to be approximately 50 mg/kg body weight/day^10^. The mean resveratrol level at 1000 mg/day was 36.9 ± 44.7 ng/mL (range, < 1.0–215.0 ng/mL) (Fig. 5a), which is consistent with the data of Almeida et al.^27^. The administration of resveratrol to healthy adult subjects at 150 mg six times/day for 13 doses resulted in a mean plasma concentration of 63.8 ng/mL and a half-life of resveratrol of 2–5 h^27^. Resveratrol at 36.9 ng/mL is equal to 160 nM. Because 160 nM resveratrol was 1/68 of the concentration needed for twofold activation of SIRT1 enzymatic activity in vitro^28^, the activation of SIRT1 by resveratrol in the present study did not seem strong. However, other studies show the physiological effects of resveratrol even in small amounts^13,27^. The administration of resveratrol at 150 mg/day for 30 days significantly decreased plasma glucose, insulin, triglycerides and leptin in healthy obese men^29^. Using muscular biopsy, Timmers et al*.* showed that resveratrol activates AMPK activity, increases SIRT1 and PGC1α levels, and promotes mitochondrial respiration activity in the muscle^29^. Based on these findings, we suggest that resveratrol at a dose of 25–50 mg/kg body weight/day is sufficient to treat MD patients. The dosing period required to detect an effect of resveratrol seems to be more than 4 months for MD patients.

Glucocorticoids have been shown to prolong the survival of patients with DMD^5^. In the present study, glucocorticoids were treated in only one patient with DMD (DMD 12 y), who showed increased muscle strength and motor function without any adverse effects during resveratrol administration. Recently, novel methods to treat DMD have been developed. Ataluren is a stop codon readthrough drug for DMD based on nonsense mutations^3^, and is currently being evaluated in a post-approval safety study in Europe. Eteplirsen is a morpholino antisense oligomer that induces the skipping of exon 51 of the dystrophin gene^4^, and accelerated approval for this drug was obtained from the U.S. Food and Drug Administration in 2016. The use of these kinds of therapies is expected to increase, although they have some limitations^30,31^. Given its unique mode of action and relatively mild adverse effects, resveratrol could combine with glucocorticoids or recent novel medicines such as ataluren and eteplirsen.

A limitation of the present study was that it was not a randomized, double-blind, placebo-controlled trial. We could not analyse an untreated group because the number of patients was limited, and three types of MD patients with various clinical conditions were recruited. In the case of rare diseases, phase I studies for cancer and phase IIa studies for other diseases have been executed without any control^32–36^. These trials were planned because of problems in recruiting patients, non-uniformity in patients’ disease conditions, the use of an investigational drug that has never been administered in humans, no prior information on dose levels, difficulty in preparing a control group or difficulty in obtaining the agreement of patients to use a placebo. Usually, if some possibility concerning effectiveness of the drug is found in a phase IIa study, a phase IIb clinical study is carried out to identify the effect and dose of the investigational drug by a randomized and double-blind study.

We repetitively measured the MFM and QMT scores of the participants. Some studies have reported that exercises increase the motor function and muscle strength of MD patients^37^. For example, the knee flexor maximum voluntary contraction torque of the limb-girdle in Becker and facioscapulohumeral MD patients increased by 13% after a 12-week resistance training programme^38^. However, two meta-analyses of exercise interventions on muscle strength in MD patients showed no evidence of improvement in muscle strength and in endurance^39^. Because the average time required for MFM has been reported to be 36 min (range 8–75 min)^15^, measuring MFM and QMT scores once every 8 weeks in the present study was unlikely to improve motor function of the patients. Since participants are aware of exercise interventions, the two meta-analyses^39^ also suggest that placebo effects of interventions are minimal on MD patients. Moreover, average muscle strength in DMD patients treated with placebo shows similar decline with that of untreated patients during administration for 6 months^40^. Because mean QMT scores of scapula elevation and shoulder abduction increased by twofold 24 weeks after resveratrol administration (Fig. 3a,b), it was less likely that the efficacy of resveratrol was a placebo effect.

As mentioned above, our study was a phase IIa study to investigate the possibility of the use of resveratrol in MD patients. To determine whether resveratrol could provide benefit for MD patients and be used as a symptomatic medication for MDs, a phase IIb study of resveratrol in a multi-centre trial with randomized, double-blind, and parallel-group comparison design will be necessary. An evaluation of the long-term effects of resveratrol on MD patients in a uniform cohort, such as age-matched young DMD patients, would be benefical.

## Methods

### Clinical study design

This study was an open-label, single-arm, phase IIa trial. The protocol was approved by the Institutional Review Board (IRB) on May 12, 2014 and registered at the University Hospital Medical Information Network Clinical Trial Registry (UMIN-CTR Unique ID: UMIN000014836) on August 12, 2014. This trial was conducted in accordance with the Declaration of Helsinki and was carried out at Sapporo Medical University Hospital from June 2014 to September 2015. Patient inclusion criteria were as follows: (1) DMD, BMD, or FCMD diagnosed based on clinical manifestations, family history, pathological findings of muscle biopsy, and/or genetic examination; and (2) minimum age of 12 years. Patients with a history of hypersensitivity to resveratrol were excluded. The daily dose of resveratrol was 500 mg/day, which was increased every 8 weeks to 1000 and then to 1500 mg/day. During the 24 weeks of medication, motor activity and blood were examined four and seven times, respectively (Fig. 1). Echocardiography and pulmonary function tests were performed prior to administration of resveratrol and after 24 weeks of medication (Fig. 1).

### Reagents and evaluation methods

Resveratrol (TransMax) was purchased from Biotivia (New York, NY, USA). Physical examinations and blood draws were performed as shown in Fig. 1. Motor function and muscle strength were evaluated by well-trained physical therapists prior to the initiation of resveratrol administration and at 8, 16, and 24 weeks. Motor function was estimated with the MFM scale^15^. The total score was calculated by adding the individual scores of 32 items and ranged from 0 to 96. The generic grading at each item is as follows: 0, does not initiate movement or starting position cannot be maintained; 1, partially completes the exercise; 2, completes the exercise with compensations, slowness or obvious clumsiness; and 3, completes the exercise with a standard pattern. MFM total score and D1 (standing position and transfers), D2 (axial and proximal motor function), and D3 (distal motor function) sub-scores were analysed^15^. Muscle strength was evaluated by quantitative muscle testing (QMT)^16–18^. QMT was performed using a hand-held dynamometer (μTas F-1; ANIMA Co., Tokyo, Japan), squeeze dynamometer (Smedley Dynamometer TTM-YO II; Tsutsumi Co., Tokyo, Japan), and pinch strength metre (SPR-641; SAKAI Medical Co., Tokyo, Japan). Pinch, grip, scapula elevation, shoulder abduction, elbow flexion, elbow extension, hip flexion, knee extension, and ankle dorsiflexion were measured. Ejection fraction (EF), percent fractional shortening (%FS), and left ventricular end-diastolic diameter (LVDd) were measured with echocardiography. Pulmonary function tests and blood were examined at Sapporo Medical University Hospital, and resveratrol concentration was measured by liquid chromatography–tandem mass spectrometry at Toray Research Center (Tokyo, Japan). Adverse events were classified, and their degree of severity was determined according to the Common Terminology Criteria for Adverse Events (CTCAE) v.4.0.

### Statistical analysis

As the primary endpoint, the MFM score, QMT score, and CK value were evaluated as continuous variables. All data and average values at each visit were plotted. Average values at each visit were compared with those before medication and were analysed using ANOVA. Post hoc analysis was performed using Dunnett’s test. P < 0.05 was considered statistically significant. For the QMT score, participants in whom the baseline value of each item could not be measured were excluded. Statistical analyses were performed using SPSS v.22 software (IBM, Armonk, NY, USA).

### Ethics declarations

*Study approval* The protocol was approved by the IRB of Sapporo Medical University Hospital on May 12, 2014. The protocol was then slightly modified twice and approved by the IRB on Sep. 18, 2014 and Jan. 28, 2015. Written informed consent was obtained from all participants or their legal guardians.

## Supplementary information

Supplementary Information.

## Data Availability

The datasets generated during and/or analysed during the current study are available from the corresponding authors on reasonable request.
