# Supplementary material for: Resveratrol improves motor function in patients with muscular dystrophies: an open-label, single-arm, phase IIa study
Source: Sci Rep. 2020 Nov 25;10:20585. doi: 10.1038/s41598-020-77197-6 (PMC7688653; doi:10.1038/s41598-020-77197-6)
Supplement: Supplementary file 1 — Supplementary Information. [file 41598_2020_77197_MOESM1_ESM.pdf]

Supplementary information

Resveratrol improves motor function in patients with muscular dystrophies: An open-label, single-arm phase IIa study

Kentaro Kawamura[1], Shinobu Fukumura[1], Koki Nikaido[1], Nobutada Tachi[2], Naoki Kozuka[3], Tsugumi Seino[3], Kingya Hatakeyama[1], Mitsuru Mori[2], Yoichi M. Ito[4], Akiyoshi Takami[5], Shiro Hinotsu[6], Atsushi Kuno[7], Yukihiko Kawasaki[1], Yoshiyuki Horio[7]*, Hiroyuki Tsutsumi[1]*

[1]Departments of Pediatrics, Sapporo Medical University School of Medicine, Sapporo 060-8543, Japan.

[2]Faculty of Health Science, Hokkaido Chitose College of Rehabilitation, Chitose 066-0055, Japan.

[3]Department of Physical Therapy, Sapporo Medical University School of Health Sciences, Sapporo 060-8556, Japan.

[4]Research Center for Medical and Health Data Science, The Institute of Statistical Mathematics, Tachikawa 190-8562, Japan.

[5]Division of Hematology, Department of Internal Medicine, Aichi Medical University School of Medicine, Nagakute 480-1195, Japan.

[6]Department of Biostatistics, Sapporo Medical University School of Medicine, Sapporo 060-8556, Japan.

[7]Department of Pharmacology, Sapporo Medical University School of Medicine, Sapporo 060-8556, Japan.

Supplementary Figure S1

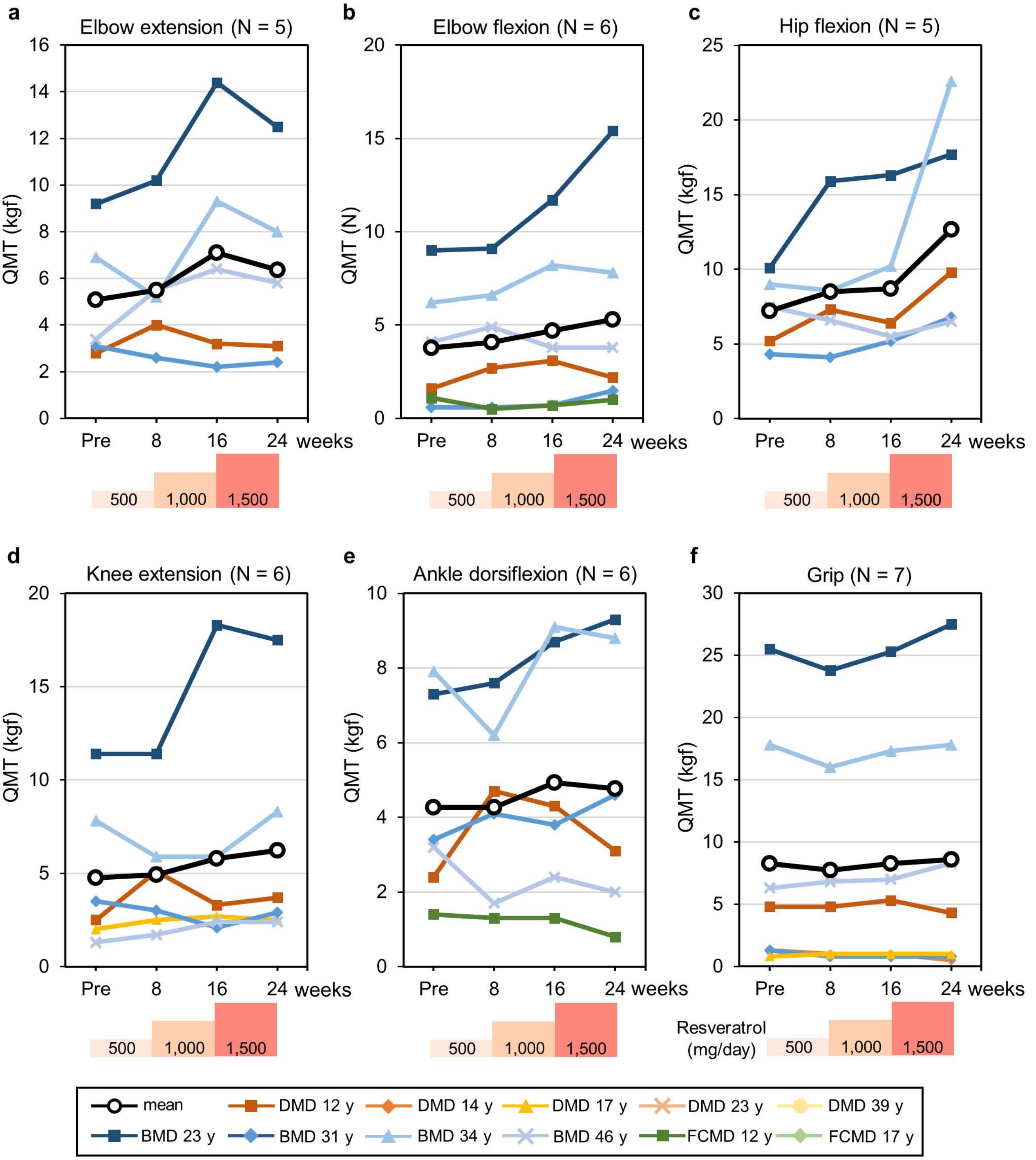

Muscle strength following resveratrol administration. (**a-f**) Time-course of muscle strength for (a) elbow extension (N = 5), (**b**) elbow flexion (N = 6), (**c**) hip flexion (N = 5), (**d**) knee extension (N = 6), (**e**) ankle dorsiflexion (N = 6) and (**f**) grip (N = 7) measured by QMT.

Supplementary Table S1. Blood tests, echocardiographic parameters and pulmonary function in each patient.

DMD 12 y

Weeks	Pre	4	8	12	16	20	24
WBC, ×10^3/μl	6400	8900	6700	8100	7300	6500	6800
Hb, mg/dl	14.1	13.3	13.3	14.3	13.8	13.2	13.1
Plt, ×10^3/μl	396	368	348	357	384	365	374
CK, unit/l	9518	5850	5161	7130	10247	4459	5167
AST, unit/l	141	95	77	104	145	66	84
ALT, unit/l	242	156	142	179	225	128	150
LDH, unit/l	702	533	457	608	712	444	488
γ-GTP, mg/dl	16	13	13	18	17	14	20
T.Bil, mg/dl	0.4	0.6	0.5	0.5	0.4	0.5	0.4
D.Bil, mg/dl	0.1	0.1	0.1	0.1	0.1	0.1	0.1
BUN, mg/dl	8	9	10	11	8	7	9
Cre, mg/dl	0.15	0.14	0.13	0.13	0.12	0.13	0.15
Cys-C, mg/l	0.72		0.83		0.59		0.78
TG, mg/dl	8	150	117	206	177	195	204
T.Cho, mg/dl	208	178	190	201	203	171	205
Na, mEq/l	143	142	140	141	141	141	141
K, mEq/l	4.5	3.8	3.7	4.4	4.2	3.6	4.1
Cl, mEq/l	104	106	105	104	103	104	105
BNP, pg/ml	15.8	10.1	9.9	12.6	15.8	15.1	23.4
EF, %	73.0						60.5
%FS, %	35.4						26.8
LVDd, mm	41.8						38.2
VC, ml	1780						1680
%VC, %	86.8						82.0
PCF, l/min	275						240

ALT, Alanine aminotransferase; AST, Aspartate aminotransferase; BNP, Brain natriuretic peptide; BUN, Blood urea nitrogen; CK, Creatine kinase; Cl, Chlorine; Cre, Creatinine; Cys-C, Cystatin-C; D.Bil, Direct bilirubin; EF, Ejection fraction; %FS, %Fractional shortening; γ-GTP, γ-glutamyl transpeptidase; Hb, Haemoglobin; K, PotassiumlDH, Lactate dehydrogenase; LVDd, Left ventricular end-diastolic diameter; Na, Sodium; PCF, Peak cough flow; Plt, Platelet; T.Bil, Total bilirubin; T.Cho Total cholesterol; TG, Triglycerides; VC, Vital capacity; WBC, White blood cell.

DMD 14 y

Week	Pre	4	8	12	16	20	24
WBC, ×10^3/μl	3400	3900	3700	3500	3800	3300	3100
Hb, mg/dl	14.1	14	13.9	13.3	13.8	13.4	14
Plt, ×10^3/μl	225	249	246	269	261	230	237
CK, unit/l	1250	1692	1982	1285	1744	1975	1739
AST, unit/l	32	38	38	33	42	43	41
ALT, unit/l	27	34	30	28	35	34	33

LDH, U/l	231	268	246	245	279	276	285
γ-GTP, mg/dl	9	10	10	9	10	9	11
T.Bil, mg/dl	0.8	0.7	0.8	0.7	0.4	0.5	0.7
D.Bil, mg/dl	0.2	0.2	0.2	0.2	0.1	0.1	0.2
BUN, mg/dl	6	8	7	7	7	6	7
Cre, mg/dl	0.06	0.07	0.15	0.05	0.05	0.06	0.06
Cys-C, mg/l	0.62		0.64		0.65		0.63
TG, mg/dl	41	61	70	68	105	80	83
T.Cho, mg/dl	171	187	181	185	187	175	181
Na, mEq/l	138	139	140	138	139	139	139
K, mEq/l	3.8	2.9	4.1	4	3.9	4	3.9
Cl, mEq/l	102	103	103	102	102	104	101
EF, %	63.1						57.4
%FS, %	28.3						24.7
LVDd, mm	54.3						45.2
VC, ml	1180						1330
%VC, %	31.5						34.7
PCF, l/min	220						210

DMD 17 y

Week	Pre	4	8	12	16	20	24
WBC, ×10^3/μl	5300	5000	5200	4300	4400	5000	4800
Hb, mg/dl	15.5	15	15	14.8	15.1	14.6	14.9
Plt, ×10^3/μl	353	352	249	30	352	324	335
CK, unit/l	1098	1171	842	887	877	878	1396
AST, unit/l	31	34	28	28	29	31	32
ALT, unit/l	53	53	41	34	39	44	36
LDH, unit/l	274	238	293	272	229	240	251
γ-GTP, mg/dl	22	17	18	15	15	16	12
T.Bil, mg/dl	0.6	1	0.6	1	0.9	0.8	0.8
D.Bil, mg/dl	0.2	0.3	0.2	0.3	0.3	0.2	0.2
BUN, mg/dl	10	11	11	10	10	8	10
Cre, mg/dl	0.06	0.04	0.06	0.05	0.05	0.07	0.05
Cys-C, mg/l	0.75		0.79		0.76		0.78
TG, mg/dl	115	59	75	86	75	88	63
T.Cho, mg/dl	135	141	150	145	165	172	164
Na, mEq/l	140	142	143	140	140	142	144
K, mEq/l	4.4	4.1	3.9	4.1	4.3	3.7	4.1
Cl, mEq/l	102	106	105	103	103	104	107
BNP, pg/ml	<0.4	4	<0.4	4.2	5.3	5.6	5.8
EF, %	44.7						42.7
%FS, %	18.0						16.7
LVDd, mm	50.8						49.8
VC, ml	680						600
%VC, %	16.3						14.4
PCF, l/min	170						160

DMD 23 y

Week	Pre	4	8	12	16	20	24
WBC, ×10^3/μl	4600	5800	5300	4300	4400	4200	6000
Hb, mg/dl	16.1	14.1	14.6	14.9	15.1	14.7	15.3
Plt, ×10^3/μl	248	367	239	209	250	307	226
CK, unit/l	546	246	473	662	412	449	400
AST, unit/l	38	20	48	41	34	33	32
ALT, unit/l	40	27	51	43	3	34	29
LDH, unit/l	203	246	184	202	228	186	201
γ-GTP, mg/dl	32	37	40	26	34	45	30
T.Bil, mg/dl	0.5	0.4	0.6	0.5	0.6	0.4	0.5
D.Bil, mg/dl	0.1	0.1	0.1	0.1	0.1	0.1	0.1
BUN, mg/dl	13	8	10	10	9	9	10
Cre, mg/dl	0.06	0.06	0.07	0.04	0.07	0.06	0.07
Cys-C, mg/l	0.77		0.82		0.74		0.68
TG, mg/dl	70	73	77	67	72	85	60
T.Cho, mg/dl	146	127	161	157	158	155	148
Na, mEq/l	141	138	141	140	138	141	141
K, mEq/l	4.4	4.7	4.5	4.7	4.5	4.5	4.3
Cl, mEq/l	105	105	107	104	103	105	105
BNP, pg/ml	<0.4	<0.4	6.8	4	<0.4	<0.4	<0.4
EF, %	63.0						NA
%FS, %	28.2						NA
LVDd, mm	54.2						NA
VC, ml	550						610
%VC, %	13.9						15.4
PCF, l/min	90						100

DMD 39 y

Week	Pre	4	8	12	16	20	24
WBC, ×10^3/μl	6900	10800	6700	6500	6800	7900	6800
Hb, mg/dl	15.6	14	14.9	13.8	14.5	15	13.6
Plt, ×10^3/μl	277	280	302	286	280	285	336
CK, unit/l	200	155	178	177	248	268	166
AST, unit/l	30	25	31	26	36	31	34
ALT, unit/l	36	33	39	36	41	38	46
LDH, unit/l	178	195	168	140	209	180	163
γ-GTP, mg/dl	33	28	29	28	28	31	37
T.Bil, mg/dl	0.5	0.5	0.8	0.7	0.5	0.6	0.4
D.Bil, mg/dl	0.1	0.1	0.2	0.2	0.1	0.2	0.1
BUN, mg/dl	11	12	8	8	8	12	10
Cre, mg/dl	0.06	0.04	0.04	0.03	0.02	0.04	0.06
Cys-C, mg/l	0.75		0.74		0.74		0.69
TG, mg/dl	106	106	131	134	101	134	178
T.Cho, mg/dl	138	136	170	147	161	172	163
Na, mEq/l	140	140	140	140	138	139	140
K, mEq/l	4.2	4.5	4	3.7	4.1	3.8	4.1
Cl, mEq/l	106	107	106	107	106	106	107
BNP, pg/ml	17.3	23.7	16.3	17	15.8	17.7	15.7
EF, %	37.4						37.9

%FS, %	14.5						14.7
LVDd, mm	58.0						55.8
VC, ml	NA						NA
%VC, %	NA						NA
PCF, l/min	NA						NA

BMD 23 y

Week	Pre	4	8	12	16	20	24
WBC, ×10^3/μl	4400	6300	5600	3900	5000	5400	4800
Hb, mg/dl	14.8	14.2	14.5	14.3	14.8	14.7	14.1
Plt, ×10^3/μl	199	263	245	239	236	247	229
CK, unit/l	431	423	493	821	754	988	316
AST, unit/l	25	24	25	32	33	47	21
ALT, unit/l	19	24	17	19	27	29	16
LDH, unit/l	265	208	213	191	199	257	213
γ-GTP, mg/dl	14	17	18	16	16	15	17
T.Bil, mg/dl	0.8	0.9	0.5	0.6	0.7	0.6	0.8
D.Bil, mg/dl	0.2	0.3	0.1	0.1	0.2	0.2	0.2
BUN, mg/dl	12	10	12	12	9	9	9
Cre, mg/dl	0.43	0.43	0.52	0.43	0.44	0.47	0.43
Cys-C, mg/l	0.75		0.83		0.79		0.75
TG, mg/dl	50	54	82	66	66	71	48
T.Cho, mg/dl	146	145	154	151	161	167	135
Na, mEq/l	140	140	142	141	140	139	139
K, mEq/l	4.2	4	4	3.7	4	4.1	3.9
Cl, mEq/l	106	106	108	107	107	105	107
BNP, pg/ml	5.6	8.3	4.2	3.7	6	6.6	14.6
EF, %	75.9						77.4
%FS, %	37.8						39.2
LVDd, mm	53.3						54.9
VC, ml	4520						4620
%VC, %	103.2						104.1
PCF, l/min	500						520

BMD 31 y

Week	Pre	4	8	12	16	20	24
WBC, ×10^3/μl	5900	5100	5100	4700	5200	5200	5400
Hb, mg/dl	15.9	14.4	15.4	14.8	15.2	15.3	15.3
Plt, ×10^3/μl	245	253	273	255	253	252	266
CK, unit/l	2401	949	988	423	902	721	714
AST, unit/l	61	39	37	24	32	32	32
ALT, unit/l	60	59	58	40	41	47	47
LDH, unit/l	246	232	290	191	221	215	241
γ-GTP, mg/dl	14	16	13	14	14	14	15
T.Bil, mg/dl	0.7	0.9	1	1.1	0.8	0.9	0.8
D.Bil, mg/dl	0.2	0.2	0.3	0.3	0.2	0.3	0.2
BUN, mg/dl	9	7	8	7	7	8	6
Cre, mg/dl	0.15	0.12	0.17	0.14	0.21	0.13	0.15

Cys-C, mg/l	0.71		0.77		0.8		0.74
TG, mg/dl	83	98	113	99	128	103	151
T.Cho, mg/dl	143	163	153	171	156	166	167
Na, mEq/l	143	140	142	142	145	141	143
K, mEq/l	3.6	4.1	3.5	4.2	3.5	3.9	3.8
Cl, mEq/l	106	106	107	106	108	105	107
BNP, pg/ml	12.3	5	4.3	6.1	15	9.3	7
EF, %	68.4						64.0
%FS, %	31.9						28.8
LVDd, mm	48.8						53.2
VC, ml	4180						4310
%VC, %	99.5						102.6
PCF, l/min	470						430

BMD 34 y

Week	Pre	4	8	12	16	20	24
WBC, ×10^3/μl	4100	3500	5400	3300	4100	4500	5700
Hb, mg/dl	16.7	16.1	16.2	16	15.6	16.5	15.7
Plt, ×10^3/μl	250	254	258	223	271	252	233
CK, unit/l	2807	1951	1641	1850	2099	1982	2228
AST, unit/l	91	62	57	68	65	68	70
ALT, unit/l	122	116	93	113	84	102	115
LDH, unit/l	414	403	369	359	328	373	483
γ-GTP, mg/dl	23	24	23	29	24	25	20
T.Bil, mg/dl	0.7	0.8	0.5	0.6	0.5	0.6	0.7
D.Bil, mg/dl	0.1	0.2	0.1	0.1	0.1	0.1	0.2
BUN, mg/dl	16	10	12	10	11	15	9
Cre, mg/dl	0.39	0.4	0.39	0.39	0.42	0.57	0.43
Cys-C, mg/l	0.71		0.73		0.75		0.74
TG, mg/dl	122	136	182	184	293	204	157
T.Cho, mg/dl	151	161	167	168	164	179	150
Na, mEq/l	139	137	139	141	138	140	140
K, mEq/l	4.1	4	4.2	4.1	4.3	4.3	4
Cl, mEq/l	102	103	105	103	103	104	105
BNP, pg/ml	<4.0	<4.0	<4.0	6.3	4.6	<4.0	<4.0
EF, %	68.3						58.4
%FS, %	31.9						25.3
LVDd, mm	51.0						52.6
VC, ml	3070						3160
%VC, %	73.6						77.2
PCF, l/min	525						620

BMD 46 y

Week	Pre	4	8	12	16	20	24
WBC, ×10^3/μl	4800	3800	4000	4000	4500	4500	3700
Hb, mg/dl	17.4	16.7	16.4	16.1	16.4	16.6	16.5
Plt, ×10^3/μl	192	131	207	213	229	212	206
CK, unit/l	766	946	937	822	582	405	660

AST, unit/l	44	47	39	46	36	34	41
ALT, unit/l	111	112	87	98	82	87	97
LDH, unit/l	351	294	300	308	262	262	255
γ-GTP, mg/dl	64	49	44	43	52	49	49
T.Bil, mg/dl	0.6	0.8	0.8	0.7	0.7	0.7	0.7
D.Bil, mg/dl	0.1	0.2	0.2	0.1	0.1	0.1	0.1
BUN, mg/dl	10	8	9	10	12	10	9
Cre, mg/dl	0.25	0.28	0.28	0.25	0.27	0.3	0.28
Cys-C, mg/l	0.7		0.66		0.78		0.75
TG, mg/dl	268	263	360	326	371	327	278
T.Cho, mg/dl	239	230	223	221	246	248	222
Na, mEq/l	141	141	143	141	143	141	142
K, mEq/l	4.4	4.2	4.2	4.2	4.3	4.3	4.1
Cl, mEq/l	106	106	106	106	106	105	104
BNP, pg/ml	5.9	<4.0	<4.0	<4.0	<4.0	<4.0	<4.0
EF, %	75.8						83.4
%FS, %	37.7						45.1
LVDd, mm	41.9						42.4
VC, ml	3070						2860
%VC, %	83.4						79.4
PCF, l/min	450						730

FCMD 12 y

Week	Pre	4	8	12	16	20	24
WBC, ×10^3/μl	5000	5300	5600	6000	7100	7400	4900
Hb, mg/dl	13.9	13.2	13.4	13	13.2	13	14
Plt, ×10^3/μl	299	355	263	271	355	308	286
CK, unit/l	840	601	576	808	580	842	781
AST, unit/l	25	21	22	27	19	25	28
ALT, unit/l	18	14	15	19	14	17	23
LDH, unit/l	289	221	254	265	201	238	259
γ-GTP, mg/dl	12	12	13	17	16	14	17
T.Bil, mg/dl	0.3	0.3	0.4	0.4	0.3	0.4	0.4
D.Bil, mg/dl	0.1	0	0.1	0.1	0.1	0.1	0.1
BUN, mg/dl	4	4	5	7	6	6	7
Cre, mg/dl	0.04	0.03	0.05	0.07	0.04	0.07	0.04
Cys-C, mg/l	0.67		0.66		0.61		0.71
TG, mg/dl	95	137	105	88	59	85	63
T.Cho, mg/dl	175	173	169	170	157	182	189
Na, mEq/l	141	141	140	140	140	139	140
K, mEq/l	4.4	4	4.3	4	4.3	4.4	4.3
Cl, mEq/l	105	107	105	105	104	104	105
BNP, pg/ml	9.5	6.3	6.4	<4.0	5.5	<4.0	11
EF, %	72.2						70.3
%FS, %	34.7						33.3
LVDd, mm	40.3						40.6
VC, ml	610						640
%VC, %	37.9						32.0
PCF, l/min	125						145

FCMD 17 y

Week	Pre	4	8	12	16	20	24
WBC, ×10^3/μl	3800	4100	4400	3800	3500	3400	3300
Hb, mg/dl	15.3	14.6	15.3	15.2	15	14.8	14.3
Plt, ×10^3/μl	248	407	309	250	199	243	238
CK, unit/l	1981	1120	1295	1220	1177	888	902
AST, unit/l	32	25	27	29	27	27	27
ALT, unit/l	15	17	14	14	16	14	15
LDH, unit/l	367	326	309	296	285	282	283
γ-GTP, mg/dl	13	17	12	10	9	9	11
T.Bil, mg/dl	0.5	0.5	0.5	0.5	0.6	0.7	0.8
D.Bil, mg/dl	0	0.1	0.1	0.1	0.2	0.2	0.2
BUN, mg/dl	11	11	9	7	13	9	12
Cre, mg/dl	0.05	0.06	0.08	0.04	0.05	0.05	0.05
Cys-C, mg/l	0.71		0.71		0.65		0.63
TG, mg/dl	69	103	67	34	40	49	51
T.Cho, mg/dl	205	217	213	219	217	224	22
Na, mEq/l	142	145	141	141	142	141	141
K, mEq/l	4.5	4.6	4	4.4	4.1	4.3	4.2
Cl, mEq/l	106	104	106	104	104	103	105
BNP, pg/ml	7.6	7.1	6.1	6.4	4.7	4.2	6
EF, %	70.5						61.0
%FS, %	33.3						26.9
LVDd, mm	36.6						36.0
VC, ml	1000						940
%VC, %	27.4						25.8
PCF, l/min	230						170
